# 

**Published:** 1903-09-12

**Authors:** 


### NOTICE TO CORRESPONDENTS.

All MSS., Letters, Books for Review, and other matters intended for the Editor should be addressed The Editor, "The Hospital," The Hospital Buildings, 28 & 29 Southampton Street, Strand, W.C.

The Editor cannot undertake to return rejected MS., even when accompanied by stamped directed envelope.

**Notice to Advertisers and Subscribers.**

All Advertisements, Orders for Copies of The Hospital, and Business Communications should be addressed to **THE MANAGER, (Not to the Editor)**, The Hospital, 28 & 29 Southampton Street, Strand, London, W.C.

The Cost of Subscription, post free, is as follows.—Per week, 3½d.; per quarter, 3s. 9d.; per half-year, 7s. 6d.; per annum, 15s. Foreign and Colonial:—Per annum, 19s. 6d., payable, in all cases, in advance.

**NOTICE.—Vol. XXXIII. of "The Hospital" (October 1902 to March 1903) in handsome cloth binding, will shortly be on sale at the office of this Journal, price 7s. 6d. Cases for binding the Numbers contained in this Volume 1s. 6d. Index to Vol. XXXIII., 2½d., post free.**

**The monthly part for September is now ready. Price 1s., by post 1s. 3d.**

### BOOKS RECEIVED.

G. Philip and Son, Limited.

"Philip's Anatomical Model of the Female Human Body."

John Long.

"How to Take Care of a Consumptive." By M. Forrest Williams.

Longmans and Co.

"Rural Hygiene." Third Edition. By G. V. Poore, M.D.

Sampson Low, Marston and Co.

"Radium and Other Radio-Active Substances." By Wm. J. Hammer.

His Majesty's Stationery Office.

"Thirty-first Annual Report of the Local Government Board, 1901–02."

***JUST PUBLISHED.***

A Practical and up-to-date Guide

TO

### SURGICAL BANDAGING & DRESSINGS.

BY

**WM. JOHNSON SMITH, F.R.C.S.**

Principal Medical Officer Seamen's Hospital, Greenwich.

**2/-** *POST FREE*

**2/-** *POST FREE*

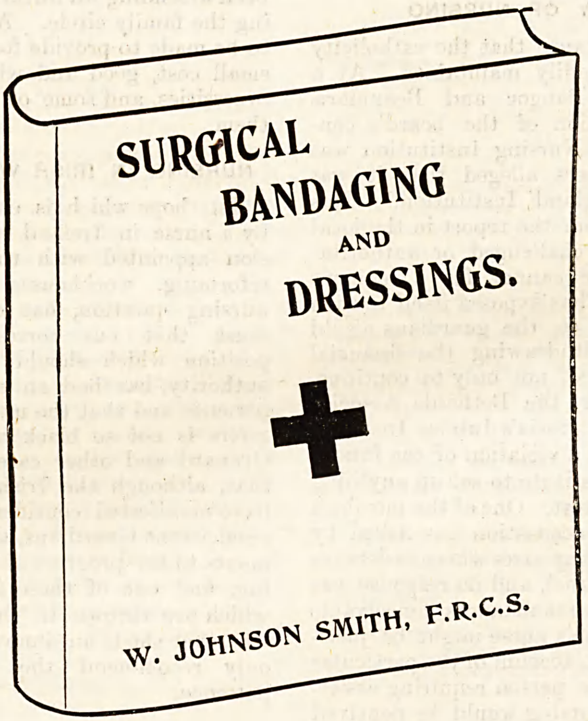

A Valuable Text-book for the Probationer.

An Indispensable Companion to the Surgical Nurse.

**2/-** *POST FREE.*

**2/-** *POST FREE.*

Actual size $3\frac{1}{2}$ inches by $4\frac{3}{4}$ inches.

BOUND IN SCARLET CLOTH BOARDS.

*Of all Booksellers, or of*

THE SCIENTIFIC PRESS, LIMITED,

28 & 29 Southampton Street, Strand, London, W.C.

